# Real-world evidence of autoimmune hepatitis following COVID-19 vaccination: A population-based pharmacovigilance analysis

**DOI:** 10.3389/fphar.2023.1100617

**Published:** 2023-04-13

**Authors:** Congqin Chen, Danfei Xie, Jie Xiao

**Affiliations:** ^1^ Department of Pharmacy, Xiamen Cardiovascular Hospital of Xiamen University, School of Medicine, Xiamen University, Xiamen, China; ^2^ Department of Medical, Songbai Hospital, Xiamen Social Welfare Center, Xiamen, China

**Keywords:** COVID-19 vaccine, autoimmune hepatitis, VAERS, CDC COVID data tracker, disproportionality analysis

## Abstract

**Objective:** Autoimmune hepatitis (AIH) has occasionally been reported after administration of Coronavirus Disease 2019 (COVID-19) vaccine. The present study aimed to investigate the reported rate and disproportionality of AIH following COVID-19 vaccination.

**Methods:** The Centers for Disease Control (CDC) COVID Data Tracker and the Vaccines Adverse Event Reporting System (VAERS) were queried between 11 December 2020 and 15 March 2022. Reported rates were calculated by cases of AIH divided by the number of vaccinated people. Disproportionate pattern of AIH for COVID-19 vaccination was accessed based on the reporting odds ratio and empirical bayes geometric mean (ROR and EBGM, respectively).

**Results:** A total of 53 reports of AIH were identified after administration of COVID-19 vaccine during the study period. The overall reported rate of COVID-19 vaccination-related AIH was 0.21 (95% CI 0.16-0.27) per million people. The results found no disproportionate reporting of AIH following COVID-19 vaccination in the VAERS (overall: ROR 1.43, 95% CI 0.52–3.96; EBGM05 0.37. mRNA: ROR 1.42, 95% CI 0.51–3.94; EBGM05 0.37. Virus vector: ROR 1.57, 95% CI 0.42–5.85; EBGM05 0.34).

**Conclusion:** COVID-19 vaccine did not increase the risk of AIH. The number of AIH cases reported to VAERS does not suggest a safety concern attributable to COVID-19 vaccine at this time.

## Introduction

The spread of Coronavirus Disease 2019 (COVID-19) has imposed a heavy burden on public health as well as global economies (Kannan et al., 2020; Salian et al., 2021). Vaccination is significantly essential to manage the COVID-19 pandemic (Hodgson et al., 2021; Soleimanpour and Yaghoubi, 2021). With the increasing number of COVID-19 vaccine given, anecdotal reports of autoimmune hepatitis (AIH) after COVID-19 vaccination are rapidly emerging from the hepatology community (Bril et al., 2021; Garrido et al., 2021; McShane et al., 2021; Vuille-Lessard et al., 2021; Camacho-Dominguez et al., 2022; Chow et al., 2022; Kang et al., 2022; Zin Tun et al., 2022). The concerns about whether COVID-19 vaccine could lead to an increase in the risk of AIH have been raised thereupon, then along comes the hesitancy to receive COVID-19 vaccination.

No population-based study addressing this doubt has been performed to date. Thus, to further evaluate whether COVID-19 vaccine was associated with AIH, a population-based study based on the Center for Disease Control and Prevention (CDC) COVID Data Tracker and the Vaccines Adverse Event Reporting System (VAERS) was conducted to investigate the reported rate and disproportionality of AIH following COVID-19 vaccination.

## Methods

### Data source

VAERS is a US system for reporting Adverse Events Following Immunization (AEFIs) that is co-administered by the CDC and the Food and Drug Administration (FDA) (Shimabukuro et al., 2015; Su et al., 2021). VAERS accepts reports from vaccine manufacturers, healthcare providers, vaccine recipients, and others. The VAERS reports include information concerning age, sex, administered vaccines, dose and lot number, post-vaccination adverse events (AEs), and health history. Signs and symptoms of AEs are coded by trained personnel using the Medical Dictionary for Regulatory Activities (MedDRA), a clinically validated, internationally standardized terminology (Shimabukuro et al., 2015; Team and Food, 2021). VAERS can be applied to detect unexpected patterns of AEFIs which are unlikely to be detected in clinical trials because of the limited number of participating vaccine recipients (Mouchet and Begaud, 2018; Bonaldo et al., 2019; Neha et al., 2020; Sato et al., 2021; Sessa et al., 2021). The CDC COVID Data Tracker is another data source used in this study. It provides comprehensive information on COVID-19 vaccination in the US, including delivered and administered doses, the number of people who received at least one dose, number of people who are fully vaccinated, and number of people who received booster dose (Lv et al., 2021).

### Data extraction

VAERS data (from 11 December 2020 to 15 March 2022) were downloaded from the website. Raw VAERS data were managed locally using the Microsoft Access software (version 2021 × 32). Each report was classified based on the following binomial factors: 1) “with” or “without” exposure to the administration of vaccines of interest (namely, COVID-19 vaccine) and 2) “with” or “without” the development of an AEFI category of interest, which was defined by combining the MedDRA 24.1 preferred terms (PTs) of “Immune-mediated cholangitis " or “Immune-mediated hepatic disorder” or “Lupoid hepatic cirrhosis” or “Autoimmune hepatitis” or “Immune-mediated hepatitis” or “Lupus hepatitis” or “Anti-liver cytosol antibody type 1 positive”. Each narrative AIH report and laboratory results were reviewed. Data, such as age, sex, dose, seriousness, and AE onset interval (from vaccination date to the reported onset of first symptoms) were also collected. The total doses of COVID-19 vaccine by administered and number of people vaccinated were acquired through the CDC COVID Data Tracker.

### Data analysis

**FIGURE 1:**
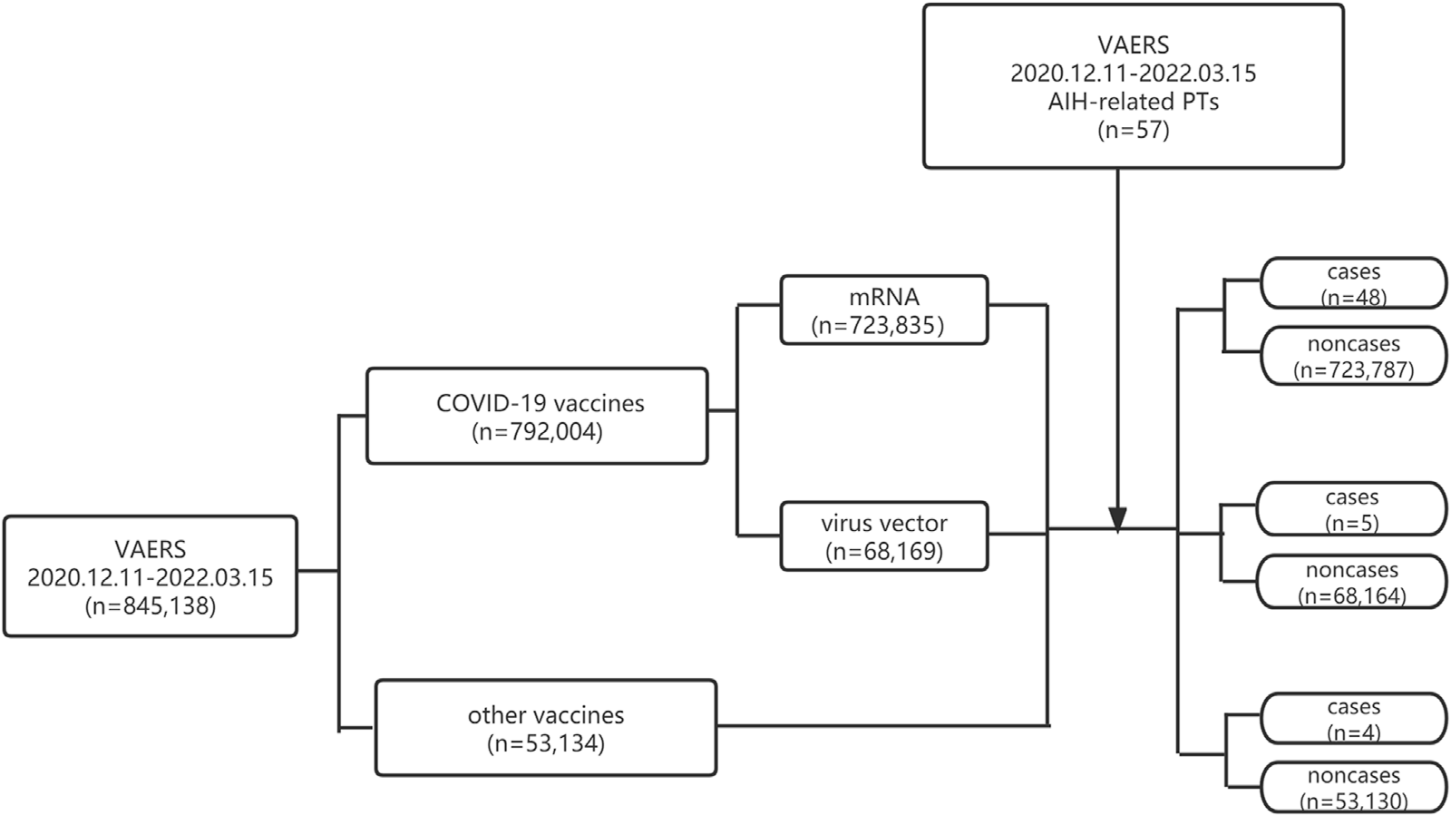
Flowchart of identifying cases and non-cases from VAERS database.

The reported rate of AIH was estimated using reports of AIH divided by the number of vaccinated people during the same study period. A population-based pharmacovigilance analysis using a case/non-case approach was performed to access the risk of AIH after COVID-19 vaccination. This system is a common approach used in pharmacovigilance studies to identify safety signals (Montastruc et al., 2011; Sato et al., 2021; Sessa et al., 2021). From the mathematical point of view, the idea of the case/non-case approach is to compare the proportion of an AE of interest in people exposed to a specific vaccine (cases) with the reports of the same reaction in people who were not exposed to this vaccine (non-cases) (Rothman et al., 2004; Sakaeda et al., 2013). This so-called case/non-case approach can be considered a case-control analysis. In our study, disproportionality was accessed by the proportional reporting ratio (ROR) and the Empirical Bayes Geometric Mean (EBGM) from Multi-Item Gamma Poisson Shrinker (MGPS). The ROR is the odd of a certain event occurring with a specific vaccine, compared to the odds of the same event occurring with all other vaccines. The ROR = (ad/cb) in which a is the number of reports of AIH for COVID-19 vaccine, b represents the reports for COVID-19 vaccine without reporting AIH, c is the number of the reports of AIH for all other vaccines, d represents the number of the reports for all other vaccines without reporting AIH (Hosohata et al., 2019). A signal emerged if the lower limits of the 95% confidence intervals (95% CI) of ROR exceeded 1 in at least three records (Zhai et al., 2022). EBGM = a (a+b + c + d)/(a + c)/(a + b). Signal was defined when the EBGM05 metric, a lower one-sided 95% confidence limit of the EBGM ≥2.0 (Sakaeda et al., 2013). The flowchart of identifying cases and non-cases from the VAERS database is shown in Figure 1.

## Results

### Reported rate of COVID-19 vaccine-related AIH

As of 15 March 2022, 792,004 AEFIs (723,835 cases for mRNA and 68,169 cases for virus vector) were found to be related to COVID-19 vaccine, and 57 reports related to AIH were documented in the VAERS database during the analytical period. The reported rates of AIH are summarized in Table 1. The overall reported rate was 0.21 (95% CI 0.16-0.27) per million people. For different age groups, the reported rate was the highest in older adults aged 65 years and over, at 0.27 (95% CI 0.15–0.44) per million people, it decreased to 0.23 (95% CI = 0.06–0.59) per million in adolescents and 0.19 (95% CI 0.13-0.26) in adults aged 18–64 years. There was no case of AIH reported in children aged 5–11 years.

**TABLE 1:** The reported rates of cases of AIH by COVID-19 vaccination based on vaccinated people.

Age group	Number of vaccinated people	Cases	Reported rate^a^
5–11 y	9,713,984	0	0
12–17 y	17,226,031	4	0.23 (0.06–0.59)
18–64 y	171,471,015	32	0.19 (0.13–0.26)
≥65 y	56,186,147	15	0.27 (0.15–0.44)
Unknown	83,957	2	—
In Total	254,681,134	53	0.21 (0.16–0.27)

^a^ Defined as per million vaccinees (95% CI).

### Descriptive analysis

The clinical characteristics of these vaccine recipients are presented in Table 2. Overall, cases of AIH were more common after administration of COVID-19 mRNA vaccine, especially following the second dose. For both mRNA and virus vector vaccines, cases of AIH were more likely to be reported in adult females. For COVID-19 mRNA vaccine, about 27.08% of AEs of AIH were hospitalized, 20.83% had emergency room (ER) visits, and 2.08% died. For reports of AIH following virus vector vaccine, 100% of the cases were hospitalized, 80% had ER visits, and none of the cases died. Among all the cases, 3 patient (5.66%) had a history of liver disease, and 1 patient (1.89%) had a history of both liver and autoimmune disease. One patient took acetaminophen during vaccination. One patient was on chronic statin therapy. Six patients were taking other potentially hepatotoxic medications (Hoofnagle et al., 2013).

**TABLE 2:** Characteristics of reports of AIH to the VAERS following COVID-19 vaccination.

Characteristics	Reports n (%)		
	mRNA	Virus vector	In total
In total	48	5	53
Age			
5–11 y	4 (8.33)	—	4 (7.55)
12–17 y	0 (0.00)	—	0 (0.00)
18–64 y	27 (56.25)	5 (100.00)	32 (60.38)
≥65 y	15 (31.25)	0 (0.00)	15 (28.30)
Unknown	2 (4.17)	0 (0.00)	2 (3.77)
Sex			
Male	12 (25.00)	1 (20.00)	13 (24.53)
Female	36 (75.00)	4 (80.00)	40 (75.47)
Dose			
1st	13 (27.08)	4 (80.00)	17 (32.08)
2nd	28 (58.33)	0 (0.00)	28 (52.83)
3rd	1 (2.08)	—	1 (1.89)
Unknown	*6 (12.50)*	1 (20.00)	7 (13.21)
Predisposing conditions			
History of liver disease	2 (4.17)	1 (20.00)	3 (5.66)
History of autoimmune disease	1 (2.08)	0 (0.00)	1 (1.89)
None of the above	46 (95.83)	4 (80.00)	50 (94.34)
Medications			
Acetaminophen	1 (2.08)	0 (0.00)	1 (1.89)
Statin	1 (2.08)	0 (0.00)	1 (1.89)
Other hepatotoxic medication	6 (12.50)	0 (0.00)	6 (11.32)
Seriousness			
Death	1 (2.08)	0 (0.00)	1 (1.89)
Life-threatening	7 (14.58)	3 (60.00)	10 (18.87)
Hospitalization	13 (27.08)	5 (100.00)	18 (33.96)
ER visit	10 (20.83)	4 (80.00)	14 (26.42)

### Disproportionality analysis

The results of overall disproportionality analysis are summarized in Table 3. These results revealed that AEs of AIH were not disproportionately reported after administration of COVID-19 vaccine (ROR 1.43, 95% CI 0.52–3.96; EBGM05 0.37). Both mRNA and virus vector COVID-19 vaccinations did not lead to an increasing risk of AIH (mRNA: ROR 1.42; 95% CI 0.51–3.94; EBGM05 0.37. Virus vector: ROR 1.57, 95% CI 0.42–5.85; EBGM05 0.34).

**TABLE 3:** Results of overall disproportionality analysis.

Vaccine type	Cases	EBGM05	ROR (95% CI)
mRNA	48	0.37	1.42 (0.51–3.94)
Virus vector	5	0.34	1.57 (0.42–5.85)
In total	53	0.37	1.43 (0.52–3.96)

### Time to onset of COVID-19 vaccine-related AIH

Generally, the median times to event onset of AIH was 16 (inter-quartile range [IQR] 3.25–37) days for mRNA vaccine, and 2 (IQR 0–4) days for virus vector vaccine. The times to onset following each type of COVID-19 vaccination are shown in Table 4. It can be seen from the data that most of the AEs of AIH occurred within 14 days after administration of all types of COVID-19 vaccine.

**TABLE 4:** Time to event onset of AIH following different types of COVID-19 vaccination.

Onset time (d)	Reports n (%)		
	mRNA	Virus vector	In total
0–7	20 (41.67)	4 (80.00)	24 (45.28)
8–14	2 (4.17)	1 (20.00)	3 (5.66)
15–30	11 (22.92)	0	11 (20.75)
≥31	13 (27.08)	0	13 (24.53)
Unspecified	2 (4.17)	0	2 (3.77)

## Discussion

Historically, the occurrence of AIH following vaccination is not new. Case reports have documented AIH following vaccinations such as influenza (Sasaki et al., 2018; Muratori et al., 2019), hepatitis A (Berry and Smith-Laing, 2007; van Gemeren et al., 2017), measles, mumps, rubella (MMR), typhoid, polio, and diphtheria/tetanus (Perumalswami et al., 2009). After EUAs of COVID-19 vaccine, cases of AIH following COVID-19 vaccination are rapidly reported (Bril et al., 2021; Garrido et al., 2021; McShane et al., 2021; Vuille-Lessard et al., 2021; Camacho-Dominguez et al., 2022; Chow et al., 2022; Kang et al., 2022; Zin Tun et al., 2022). Although causality cannot be proved based on the case anecdotes, constant vigilance for this phenomenon is needed. To the best of our knowledge, this study is the first real-world population-based study investigating the reported rate and disproportionality of AIH following COVID-19 vaccination by assessing reports submitted to the VAERS.

This study used the VAERS to retrieve reports of AIH following COVID-19 vaccination and the CDC COVID Data tracker to measure the number of vaccinated people during the same period. Hence, the common limitation of the passive surveillance data of unknown denominators was solved. Our results indicate that AIH represented a very rare AEFI for COVID-19 vaccination. In the general US population, the incidence rate of AIH is 6.7–20 cases per 1,000,000 person-year (Alvarez et al., 1999). Our study showed the reported rates of AIH after administration of both COVID-19 mRNA and virus vector vaccines were not higher than of the general population, demonstrating that COVID-19 vaccination might not be associated with AIH.

Data mining based on disproportionality analyses within VAERS has been widely used to detect safety signals of vaccines. Signals for inactivated influenza and typhoid and tetanus toxoid-containing vaccines have been successfully identified as described in previous studies (Iskander et al., 2006; Kamath et al., 2020). Our team conducted another pharmacovigilance study regarding the cardiovascular safety of COVID-19 vaccine (Chen et al., 2022). The CDC COVID Data Tracker and the VAERS were queried between 11 December 2020 and 15 March 2022 in the previous research too. We investigated the reported rates and risks of myocarditis/pericarditis following booster dose and primary series of COVID-19 mRNA vaccination. The results showed that the risks of myocarditis/pericarditis for booster dose of COVID-19 mRNA vaccination were lower than primary series course. In this present study, same data from the CDC COVID Data Tracker and the VAERS were queried to access hepatic safety of COVID-19 vaccine. A disproportionality assessment based on COVID-19 vaccine type was conducted to analyze whether COVID-19 vaccination was significantly associated with increased risk for AIH. The results showed that the lower limits of 95% CI of ROR and EBGM05 for all study sets are <1, indicating that COVID-19 vaccination does not increase the risk of AIH.

Females accounted for most of the cases of AIH following both mRNA and virus vector COVID-19 vaccinations in our study. Previous studies have reported female predilection of AIH in the general population (Gronbaek et al., 2014). Published studies (Ngu et al., 2010; Tunio et al., 2021) have also indicated higher incidence of AIH in elderly people aged >65 years. The incidence rate was the highest in older adults aged 65 years and over in our study too.

The pathophysiological mechanism behind COVID-19 vaccine-associated AIH is still unclear. Several hypotheses including molecular mimicry and activation of dormant autoreactive T-helper cells have been proposed. Molecular mimicry is thought to play a significant role in the development of autoimmune disease associated with other vaccines, such as vaccines for influenza and hepatitis B (Ahmed et al., 2015; Segal and Shoenfeld, 2018). Boettler and his colleagues (Boettler et al., 2022) reported a case of a 52-year-old male, presenting with bimodal episodes of acute hepatitis, each occurring 2–3 weeks after BNT162b2 mRNA vaccination. Imaging mass cytometry and flow cytometry were performed on liver biopsy tissue to identify the underlying immune correlates. T cell-dominant immune-mediated pathomechanism is thought to be associated with COVID-19 vaccination-induced AIH in this patient. The median time to first symptom onset was 16 days for mRNA vaccine in our study. This onset interval is consistent with the time course of the proposed hypotheses involving molecular mimicry and activation of T-cells. However, the median adverse event onset time for AIH was only 2 days for virus vector vaccine. In theory, this interval is too short to be consistent with the time course of the putatively involved immunopathologic reactions. There might be other potential mechanisms for COVID-19 virus vector vaccine. To be noted, our study only identified 5 cases of AIH related with COVID-19 virus vector vaccine in VAERS. The number might be insufficient to draw a conclusion. More studies are still warranted to address this question.

Regarding the contribution of this research to field, AIH has rapidly been reported after administration of COVID-19 vaccine. Reported rate and disproportionality pattern for AIH of COVID-19 vaccination were accessed based on the VAERS and CDC COVID Data tracker in this study. No disproportionate reporting of AIH following COVID-19 vaccination was found indicating that COVID-19 vaccine does not increase the risk of AIH.

Study limitations should be acknowledged. These limitations are mainly inherent to the nature of self-reporting database (Sato et al., 2021). Firstly, cases in VAERS might contain information that is incomplete and inaccurate, especially the lack of information on concomitant medications or comorbid medical histories. However, vaccine safety experts review all reports of serious AEs and perform further investigations for confirmation if needed. Secondly, AEs are usually under-reported in VAERS, which may lead to an underestimation of the actual associated risks. However, serious AEs, such as AIH examined in this study are more likely to be reported than non-serious ones. Reported rates were calculated by cases of interest divided by the number of vaccinated people. We used this method to estimate the risk of COVID-19 mRNA vaccination-induced myocarditis/pericarditis in previous study with a conclusion that higher reported rates of myocarditis and pericarditis after administration of COVID-19 mRNA vaccine were found compared with the general population (Chen et al., 2022). The reported rate of AIH is 0.21 cases per million vaccinees in the present study which is quite lower than the general population. The low reported rates still represented a significant demonstration that COVID-19 vaccination might not be associated with AIH, though we acknowledge that not all cases of COVID-19 vaccine-induced AIH might have been documented in the VAERS due to the limitation of self-reporting database. Results of disproportionality analysis backed up this assumption. Still, clinicians should be vigilant for AIH in patients who present with liver injury following vaccination. Healthcare providers are encouraged to report to VAERS any additional clinically significant AIH-related AEs following COVID-19 vaccination. At last, all reports are submitted to VAERS without assessing specific causality considering the events may be coincidental and related to other causes. This type of reporting might lead to an overestimation of the associated risks. Notwithstanding these limitations, disproportionality analysis still represents an invaluable method to monitor vaccine safety and identify novel rare signals.

In conclusion, our study found that fewer AIH cases were reported to VAERS than expected when considering the background rate of AIH and the number of individuals vaccinated. The COVID-19 vaccination does not lead to an increase in the risk of AIH compared with other vaccines. In the context of heightened vigilance and robust reporting to VAERS, the number of post-vaccination AIH cases do not generate a safety signal attributable to COVID-19 vaccination at this time.

## Data Availability

The raw data supporting the conclusion of this article will be made available by the authors, without undue reservation.

## References

[B1] Ahmed S. S. Volkmuth W. Duca J. Corti L. Pallaoro M. Pezzicoli A. (2015). Antibodies to influenza nucleoprotein cross-react with human hypocretin receptor 2. Sci. Transl. Med. 7 (294), 294ra105. 294ra105. 10.1126/scitranslmed.aab2354 26136476

[B2] Alvarez F. Berg P. A. Bianchi F. B. Bianchi L. Burroughs A. K. Cancado E. L. (1999). International autoimmune hepatitis group report: Review of criteria for diagnosis of autoimmune hepatitis. J. Hepatol. 31 (5), 929–938. 10.1016/s0168-8278(99)80297-9 10580593

[B3] Berry P. A. Smith-Laing G. (2007). Hepatitis A vaccine associated with autoimmune hepatitis. World J. Gastroenterol. 13 (15), 2238–2239. 10.3748/wjg.v13.i15.2238 17465509 PMC4146852

[B4] Boettler T. Csernalabics B. Salie H. Luxenburger H. Wischer L. Salimi Alizei E. (2022). SARS-CoV-2 vaccination can elicit a CD8 T-cell dominant hepatitis. J. Hepatol. 77 (3), 653–659. 10.1016/j.jhep.2022.03.040 35461912 PMC9021033

[B5] Bonaldo G. Vaccheri A. D'Annibali O. Motola D. (2019). Safety profile of human papilloma virus vaccines: An analysis of the US vaccine adverse event reporting system from 2007 to 2017. Br. J. Clin. Pharmacol. 85 (3), 634–643. 10.1111/bcp.13841 30569481 PMC6379209

[B6] Bril F. Al Diffalha S. Dean M. Fettig D. M. (2021). Autoimmune hepatitis developing after coronavirus disease 2019 (COVID-19) vaccine: Causality or casualty? J. Hepatol. 75 (1), 222–224. 10.1016/j.jhep.2021.04.003 33862041 PMC8056822

[B7] Camacho-Dominguez L. Rodriguez Y. Polo F. Restrepo Gutierrez J. C. Zapata E. Rojas M. (2022). COVID-19 vaccine and autoimmunity. A new case of autoimmune hepatitis and review of the literature. J. Transl. Autoimmun. 5, 100140. 10.1016/j.jtauto.2022.100140 35013724 PMC8730708

[B8] Chen C. Fu F. Ding L. Fang J. Xiao J. (2022). Booster dose of COVID-19 mRNA vaccine does not increase risks of myocarditis and pericarditis compared with primary vaccination: New insights from the vaccine adverse event reporting system. Front. Immunol. 13, 938322. 10.3389/fimmu.2022.938322 36172346 PMC9510366

[B9] Chow K. W. Pham N. V. Ibrahim B. M. Hong K. Saab S. (2022). Autoimmune hepatitis-like syndrome following COVID-19 vaccination: A systematic review of the literature. Dig. Dis. Sci. 67, 4574–4580. 10.1007/s10620-022-07504-w 35486203 PMC9052185

[B10] Garrido I. Lopes S. Simoes M. S. Liberal R. Lopes J. Carneiro F. (2021). Autoimmune hepatitis after COVID-19 vaccine - more than a coincidence. J. Autoimmun. 125, 102741. 10.1016/j.jaut.2021.102741 34717185 PMC8547941

[B11] Gronbaek L. Vilstrup H. Jepsen P. (2014). Autoimmune hepatitis in Denmark: Incidence, prevalence, prognosis, and causes of death. A nationwide registry-based cohort study. J. Hepatol. 60 (3), 612–617. 10.1016/j.jhep.2013.10.020 24326217

[B12] Hodgson S. H. Mansatta K. Mallett G. Harris V. Emary K. R. W. Pollard A. J. (2021). What defines an efficacious COVID-19 vaccine? A review of the challenges assessing the clinical efficacy of vaccines against SARS-CoV-2. Lancet Infect. Dis. 21 (2), e26–e35. 10.1016/S1473-3099(20)30773-8 33125914 PMC7837315

[B13] Hoofnagle J. H. Serrano J. Knoben J. E. Navarro V. J. (2013). LiverTox: A website on drug-induced liver injury. Hepatology 57 (3), 873–874. 10.1002/hep.26175 23456678 PMC5044298

[B14] Hosohata K. Inada A. Oyama S. Furushima D. Yamada H. Iwanaga K. (2019). Surveillance of drugs that most frequently induce acute kidney injury: A pharmacovigilance approach. J. Clin. Pharm. Ther. 44 (1), 49–53. 10.1111/jcpt.12748 30014591

[B15] Iskander J. Pool V. Zhou W. English-Bullard R. Team V. (2006). Data mining in the US using the vaccine adverse event reporting system. Drug Saf. 29 (5), 375–384. 10.2165/00002018-200629050-00002 16689554

[B16] Kamath A. Maity N. Nayak M. A. (2020). Facial paralysis following influenza vaccination: A disproportionality analysis using the vaccine adverse event reporting system database. Clin. Drug Investig. 40 (9), 883–889. 10.1007/s40261-020-00952-0 PMC7371962 32696320

[B17] Kang S. H. Kim M. Y. Cho M. Y. Baik S. K. (2022). Autoimmune hepatitis following vaccination for SARS-CoV-2 in korea: Coincidence or autoimmunity? J. Korean Med. Sci. 37 (15), e116. 10.3346/jkms.2022.37.e116 35437965 PMC9015903

[B18] Kannan S. Shaik Syed Ali P. Sheeza A. Hemalatha K. (2020). COVID-19 (Novel Coronavirus 2019) - recent trends. Eur. Rev. Med. Pharmacol. Sci. 24 (4), 2006–2011. 10.26355/eurrev_202002_20378 32141569

[B19] Lv G. Yuan J. Xiong X. Li M. (2021). Mortality rate and characteristics of deaths following COVID-19 vaccination. Front. Med. (Lausanne) 8, 670370. 10.3389/fmed.2021.670370 34055843 PMC8160119

[B20] McShane C. Kiat C. Rigby J. Crosbie O. (2021). The mRNA COVID-19 vaccine - a rare trigger of autoimmune hepatitis? J. Hepatol. 75 (5), 1252–1254. 10.1016/j.jhep.2021.06.044 34245804 PMC8264276

[B21] Montastruc J. L. Sommet A. Bagheri H. Lapeyre-Mestre M. (2011). Benefits and strengths of the disproportionality analysis for identification of adverse drug reactions in a pharmacovigilance database. Br. J. Clin. Pharmacol. 72 (6), 905–908. 10.1111/j.1365-2125.2011.04037.x 21658092 PMC3244636

[B22] Mouchet J. Begaud B. (2018). Central demyelinating diseases after vaccination against hepatitis B virus: A disproportionality analysis within the VAERS database. Drug Saf. 41 (8), 767–774. 10.1007/s40264-018-0652-4 29560597

[B23] Muratori P. Serio I. Lalanne C. Lenzi M. (2019). Development of autoimmune hepatitis after influenza vaccination; trigger or killer? Clin. Res. Hepatol. Gastroenterol. 43 (6), e95–e96. 10.1016/j.clinre.2019.02.007 30926201

[B24] Neha R. Subeesh V. Beulah E. Gouri N. Maheswari E. (2020). Postlicensure surveillance of human papillomavirus vaccine using the vaccine adverse event reporting system, 2006-2017. Perspect. Clin. Res. 11 (1), 24–30. 10.4103/picr.PICR_140_18 32154146 PMC7034135

[B25] Ngu J. H. Bechly K. Chapman B. A. Burt M. J. Barclay M. L. Gearry R. B. (2010). Population-based epidemiology study of autoimmune hepatitis: A disease of older women? J. Gastroenterol. Hepatol. 25 (10), 1681–1686. 10.1111/j.1440-1746.2010.06384.x 20880179

[B26] Perumalswami P. Peng L. Odin J. A. (2009). Vaccination as a triggering event for autoimmune hepatitis. Semin. Liver Dis. 29 (3), 331–334. 10.1055/s-0029-1233537 19676005

[B27] Rothman K. J. Lanes S. Sacks S. T. (2004). The reporting odds ratio and its advantages over the proportional reporting ratio. Pharmacoepidemiol Drug Saf. 13 (8), 519–523. 10.1002/pds.1001 15317031

[B28] Sakaeda T. Tamon A. Kadoyama K. Okuno Y. (2013). Data mining of the public version of the FDA adverse event reporting system. Int. J. Med. Sci. 10 (7), 796–803. 10.7150/ijms.6048 23794943 PMC3689877

[B29] Salian V. S. Wright J. A. Vedell P. T. Nair S. Li C. Kandimalla M. (2021). COVID-19 transmission, current treatment, and future therapeutic strategies. Mol. Pharm. 18 (3), 754–771. 10.1021/acs.molpharmaceut.0c00608 33464914

[B30] Sasaki T. Suzuki Y. Ishida K. Kakisaka K. Abe H. Sugai T. (2018). Autoimmune hepatitis following influenza virus vaccination: Two case reports. Med. Baltim. 97 (30), e11621. 10.1097/MD.0000000000011621 PMC6078681 30045302

[B31] Sato K. Mano T. Niimi Y. Toda T. Iwata A. Iwatsubo T. (2021). Facial nerve palsy following the administration of COVID-19 mRNA vaccines: Analysis of a self-reporting database. Int. J. Infect. Dis. 111, 310–312. 10.1016/j.ijid.2021.08.071 34492394 PMC8418051

[B32] Segal Y. Shoenfeld Y. (2018). Vaccine-induced autoimmunity: The role of molecular mimicry and immune crossreaction. Cell Mol. Immunol. 15 (6), 586–594. 10.1038/cmi.2017.151 29503439 PMC6078966

[B33] Sessa M. Kragholm K. Hviid A. Andersen M. (2021). Thromboembolic events in younger women exposed to Pfizer-BioNTech or Moderna COVID-19 vaccines. Expert Opin. Drug Saf. 20 (11), 1451–1453. 10.1080/14740338.2021.1955101 34264151 PMC8330010

[B34] Shimabukuro T. T. Nguyen M. Martin D. DeStefano F. (2015). Safety monitoring in the vaccine adverse event reporting system (VAERS). Vaccine 33 (36), 4398–4405. 10.1016/j.vaccine.2015.07.035 26209838 PMC4632204

[B35] Soleimanpour S. Yaghoubi A. (2021). COVID-19 vaccine: Where are we now and where should we go? Expert Rev. Vaccines 20 (1), 23–44. 10.1080/14760584.2021.1875824 33435774 PMC7898300

[B36] Su J. R. McNeil M. M. Welsh K. J. Marquez P. L. Ng C. Yan M. (2021). Myopericarditis after vaccination, vaccine adverse event reporting system (VAERS). Vaccine, 39(5):839–845. 10.1016/j.vaccine.2020.12.046 33422381

[B37] Team C. C-R. Food Drug A. (2021). Allergic reactions including anaphylaxis after receipt of the first dose of pfizer-BioNTech COVID-19 vaccine - United States, december 14-23, 2020. MMWR Morb. Mortal. Wkly. Rep. 70 (2), 46–51. 10.15585/mmwr.mm7002e1 33444297 PMC7808711

[B38] Tunio N. A. Mansoor E. Sheriff M. Z. Cooper G. S. Sclair S. N. Cohen S. M. (2021). Epidemiology of autoimmune hepatitis (AIH) in the United States between 2014 and 2019: A population-based national study. J. Clin. Gastroenterol. 55 (10), 903–910. 10.1097/MCG.0000000000001449 33074948 PMC8050120

[B39] van Gemeren M. A. van Wijngaarden P. Doukas M. de Man R. A. (2017). Vaccine-related autoimmune hepatitis: The same disease as idiopathic autoimmune hepatitis? Two clinical reports and review. Scand. J. Gastroenterol. 52 (1), 18–22. 10.1080/00365521.2016.1224379 27565372

[B40] Vuille-Lessard E. Montani M. Bosch J. Semmo N. (2021). Autoimmune hepatitis triggered by SARS-CoV-2 vaccination. J. Autoimmun. 123, 102710. 10.1016/j.jaut.2021.102710 34332438 PMC8316013

[B41] Zhai Y. Ye X. Hu F. Xu J. Guo X. Lin Z. (2022). Updated insights on cardiac and vascular risks of proton pump inhibitors: A real-world pharmacovigilance study. Front. Cardiovasc Med. 9, 767987. 10.3389/fcvm.2022.767987 35282344 PMC8913586

[B42] Zin Tun G. S. Gleeson D. Al-Joudeh A. Dube A. (2022). Immune-mediated hepatitis with the Moderna vaccine, no longer a coincidence but confirmed. J. Hepatol. 76 (3), 747–749. 10.1016/j.jhep.2021.09.031 34619252 PMC8491984

